# Investigating the correlation between tertiary lymphoid structures and clinical outcomes in pancreatic ductal adenocarcinoma: insights into tumor immunology

**DOI:** 10.3389/fonc.2025.1569947

**Published:** 2025-06-26

**Authors:** Lingbo Hu, Yanhong Xiao, Ning Jiang, Yufen Hu, Liewang Qiu, Bo Geng

**Affiliations:** ^1^ Department of Hepatobiliary Surgery, The First Affiliated Hospital of Chongqing Medical University, Chongqing, China; ^2^ Department of Hepatopancreatobiliary Surgery, Taizhou Hospital of Zhejiang Province Affiliated to Wenzhou Medical University, Taizhou, Zhejiang, China; ^3^ Department of Hepatopancreatobiliary Surgery, Enze Hospital, Taizhou Enze Medical Center, Taizhou, Zhejiang, China; ^4^ Department of Pathology, Dianjiang County Hospital of Traditional Chinese Medicine, Chongqing, China; ^5^ Molecular Medicine Diagnostic and Testing Center, Chongqing Medical University, Chongqing, China; ^6^ Department of Pathology, the First Affiliated Hospital of Chongqing Medical University, Chongqing, China; ^7^ Department of General Practice, Shiqiaopu Community Health Service Center, Chongqing, China; ^8^ Department of Gastroenterology, Affiliated Yongchuan Hospital of Chongqing Medical University, Chongqing, China; ^9^ Department of Hepatobiliary Surgery, People’s Hospital of He Chuan Chong Qing, Chongqing, China

**Keywords:** tertiary lymphoid structures, pancreatic ductal adenocarcinoma, immune regulation, prognostic biomarker, overall survival

## Abstract

Pancreatic ductal adenocarcinoma (PDAC) is associated with poor prognosis and high mortality rates, necessitating the identification of reliable biomarkers for patient stratification. This study investigates the prognostic significance and biological functions of tertiary lymphoid structures (TLS) in PDAC. We analyzed clinical data from 65 PDAC patients and RNA sequencing data from The Cancer Genome Atlas (TCGA) to evaluate the presence of TLS and its impact on overall survival (OS) and recurrence-free survival (RFS). Our results demonstrate that patients with intratumoral TLS (iTLS+) exhibit significantly improved OS and RFS compared to those without iTLS (iTLS-). Additionally, the presence of TLS correlates with enhanced immune cell infiltration, including increased levels of B cells, T cells, and plasma cells, suggesting a more active anti-tumor immune response in the TLS+ group. Furthermore, we found significant differences in gene expression between TLS+ and TLS- groups, particularly in pathways related to tumor proliferation and immune regulation. Notably, our findings indicate that the mutation frequency of key oncogenes, such as TP53, differs significantly between these groups, potentially influencing patient outcomes. This study underscores the potential of TLS as a prognostic biomarker in PDAC and highlights their role in modulating the tumor microenvironment. Our findings pave the way for further research into TLS-targeted therapeutic strategies aimed at improving the clinical management of pancreatic cancer.

## 1 Introduction

Pancreatic ductal adenocarcinoma (PDAC) is the most common subtype of pancreatic cancer and holds the position of twelfth in incidence and sixth in mortality on a global scale (1). Despite notable progress in surgical methods, chemotherapy, and radiation therapy, the outlook for PDAC patients remains grim, with a five-year survival rate falling below 10% (2). The tumor microenvironment plays a key role in cancer recurrence and metastasis.For instance, effective infiltration and activation of T cells in the tumor microenvironment (TME) are associated with significantly improved long-term survival rates in those with pancreatic cancer (3). Additionally, the arrangement and composition of immune cells within this environment have emerged as vital factors influencing tumor invasion, metastasis, and the overall prognosis of cancer patients (4–6).

Tertiary lymphoid structures (TLS) are ectopic lymphoid formations that emerge in non-lymphoid tissues as a consequence of chronic inflammation and tumorigenesis. These structures can be classified into distinct categories based on their composition of follicles and germinal centers, indicating a multi-stage maturation process: early TLS (lymphoid aggregates), primary TLS (follicles lacking germinal centers), and secondary TLS (follicles containing germinal centers) (7). The presence of TLS has been linked to improved prognostic outcomes in several cancers, such as breast cancer (8), lung cancer (9), colorectal cancer (10), hepatocellular carcinoma (11), and pancreatic ductal adenocarcinoma (PDAC) (12). Previous studies link TLS presence to better prognosis, but the tumor microenvironment in PDAC with or without TLS is still poorly understood (12–15). In this study, we utilized clinical data to validate the prognostic significance of TLS in PDAC patients, alongside RNA sequencing data from The Cancer Genome Atlas (TCGA) to investigate the differences in TME between PDAC cases with and without TLS.

## 2 Methods

### 2.1 Patients and samples

This study received ethical approval from the Ethics Committee of the First Affiliated Hospital of Chongqing Medical University. Informed consent was obtained preoperatively for the use of surgical specimens and associated clinical data. We conducted a retrospective analysis of patients diagnosed with pancreatic ductal adenocarcinoma (PDAC) at the First Affiliated Hospital of Chongqing Medical University from January 2017 to August 2023. The clinical and biological parameters recorded included age, gender, carcinoembryonic antigen (CEA) levels, carbohydrate antigen 19-9 (CA19-9) levels, disease stage (as per the 8th edition of the American Joint Committee on Cancer tumor-node-metastasis [AJCC TNM] classification system), T classification, N classification, and histological grade.

Additionally, we accessed clinical, biological, and gene expression data for pancreatic cancer from The Cancer Genome Atlas (TCGA) database (https://portal.gdc.cancer.gov/). In this TCGA cohort, we included 147 patients diagnosed with PDAC who had corresponding RNA sequencing data and 143 patients with available overall survival data for further analysis.

The primary clinical endpoints of this study were overall survival (OS) and recurrence-free survival (RFS). OS was defined as the time from the date of hepatectomy to the date of death or, for living patients, the date of the last follow-up. RFS was defined as the time from surgical resection to the first occurrence of recurrence or progression, death from any cause, or the date of the last follow-up.

### 2.2 Pathological examination

We prepared dual sections for H&E staining to assess tissue morphology and cellular features. These sections were subjected to independent evaluation by two experienced pathologists, each possessing extensive expertise in histopathological analysis. The examination of all sections was performed using a high-resolution digital imaging system with a 40x objective lens, enabling precise visualization of the tissue architecture and any intra-tumoral structures. Subsequently, the digitized images were analyzed using Motic DSAssistant software, a powerful tool designed for quantitative histopathological assessment. The primary objective of this analysis was to determine the presence and distribution of intra-tumoral tertiary lymphoid structures (iTLS), which are indicative of immune activity within the tumor microenvironment. For the purpose of interpretation, the pathologists categorized the sections based on the presence of TLS. Sections that lacked any identifiable TLS were classified as iTLS−, indicating an absence of these structures. Conversely, sections that exhibited the presence of TLS were classified as iTLS+, signifying the formation of these lymphoid aggregates within the tumor. This classification is crucial for further understanding the potential implications of iTLS in tumor biology and patient prognosis.

### 2.3 Immunohistochemistry

We conducted immunohistochemical analyses on sections containing mature tertiary lymphoid structures (TLS) to characterize the immune cell composition present within these structures. For this purpose, formalin-fixed, paraffin-embedded tumor blocks were utilized to prepare serial sections with a thickness of 4 micrometers. We performed immunohistochemistry and quantified immune cell infiltration using standard protocols (12). The slides were stained for key immune markers, including CD3, which identifies total T cells; CD4 and CD8, which differentiate between helper T cells and cytotoxic T cells, respectively; and PD-L1, a critical checkpoint protein that plays a role in regulating immune responses. This comprehensive staining approach enabled us to assess the distribution and density of various immune cell populations within the mature TLS, thereby providing valuable insights into the immune microenvironment associated with the tumor.

### 2.4 Evaluation of prognostic value

Kaplan-Meier survival analysis was performed to compare OS and RFS between PDAC patients with and without iTLS. Univariate and multivariate Cox regression analyses were utilized to determine whether specific parameters served as significant prognostic factors for PDAC. Independent prognostic factors were incorporated into a multivariate Cox proportional hazards model to predict survival outcomes in PDAC patients.

### 2.5 Analysis of gene expression differences between TLS+ and TLS- groups

RNA sequencing raw count data were processed and analyzed using the edgeR (v4.2.0) package to assess gene expression variations based on TLS status (iTLS+ versus iTLS−). Robustness was verified by filtering using filterByExpr. We applied an adjusted p-value of < 0.05, coupled with a threshold of |log2 fold change (FC)| > 1, to determine statistically significant genes. This approach allowed us to pinpoint genes that are differentially expressed and may play a crucial role in tumor biology associated with the presence of intra-tumoral TLS, providing insights into the underlying mechanisms of immune modulation in the tumor microenvironment.

### 2.6 Biological functional analysis

We employed the R package “clusterProfiler” to conduct comprehensive enrichment analyses, including Gene Ontology (GO) assessments for biological processes, cellular components, and molecular functions, as well as Kyoto Encyclopedia of Genes and Genomes (KEGG) pathway analyses. Different expression genes between TLS+ and TLS- groups were included in both the GO and KEGG analyses. In addition, we performed Gene Set Enrichment Analysis (GSEA) using both the Canonical Pathways and ontology gene sets. The genes for GSEA were ranked by the value of logFC. This analysis aimed to elucidate the signaling pathways associated with differentially expressed genes, providing insights into the biological mechanisms underlying the observed gene expression profiles. By integrating these analytic methodologies, we aimed to enhance our understanding of the molecular landscape of pancreatic ductal adenocarcinoma and identify potential therapeutic targets for intervention.

### 2.7 Analysis of immune microenvironment in TLS+ and TLS- groups

The R package “estimate” was employed to assess the tumor microenvironment of each sample within the TLS+ and TLS− groups. This analysis calculated key parameters, including the immune score, matrix score, ESTIMATE score, and tumor purity for each sample, providing a comprehensive evaluation of the tumor’s immune landscape and its associated stromal components. In addition, the R package “GSVA” was utilized for single-sample gene set enrichment analysis (ssGSEA) to visualize the differential enrichment of immune-related cell types and functional pathways across high- and low-risk groups. This method allows for a nuanced understanding of how specific immune functions vary between these groups, highlighting potential immune responses linked to tumor behavior. Furthermore, the ‘CIBERSORT’ package was used to analyze the infiltration levels of 22 distinct immune cell types within the high- and low-risk groups. This analysis facilitated a detailed comparison of immune cell composition, enabling the identification of significant differences in immune cell infiltration that may correlate with patient outcomes and the tumor microenvironment.

### 2.8 Analysis of mutation characteristics in TLS+ and TLS- groups

The R package “mafools” was employed to effectively visualize the mutation sites of high-frequency mutant genes within both high-risk and low-risk patient groups. This tool facilitated a comprehensive analysis of the genetic landscape associated with pancreatic ductal adenocarcinoma by enabling the exploration of co-mutation patterns and instances of mutual exclusion among these genes. By generating detailed mutation maps, “mafools” allowed for an intuitive comparison of genetic alterations between the distinct risk groups, thereby providing valuable insights into the potential interactions and functional implications of these mutations. Such analyses are crucial for understanding the underlying mechanisms of tumorigenesis and for identifying potential therapeutic targets in the management of pancreatic cancer.

### 2.9 Analysis of small molecule drug sensitivity in TLS+ and TLS- groups

The R package “pRRophetic” was utilized to assess sensitivity scores, thereby predicting the maximum half-inhibitory concentration (IC50) of various therapeutic agents for each patient (16). This tool leverages gene expression data to estimate the drug response profiles of individual tumors, offering insights into potential treatment efficacy. To investigate the differences in IC50 values between patients with iTLS (+) and those without iTLS (−), a t-test analysis was conducted. This statistical approach facilitated the comparison of drug sensitivity across the two groups, highlighting significant variations that may influence treatment decisions. To visually represent the distribution of IC50 values, the ‘ggplot2’ package was employed to generate a violin plot, a powerful graphical tool that illustrates both the density and distribution of the data. Through this analysis, we aimed to identify potential therapeutic agents that may be particularly effective for patients with PDAC, thus advancing personalized treatment strategies.

### 2.10 Prediction of response to immunotherapy in TLS+ and TLS- groups

The Tumor Immune Dysfunction and Exclusion (TIDE) algorithm was employed to predict the response to immunotherapy in the studied cohorts. This algorithm integrates various immune-related parameters to assess how well a tumor might respond to immune checkpoint inhibitors and other immunotherapeutic approaches. To evaluate the differential response predictions between the groups classified by the presence of iTLS, namely TLS+ and TLS-, we utilized the Wilcoxon test. This non-parametric statistical test allows for the comparison of the response predictions between the two groups, providing insights into the potential impact of iTLS on the efficacy of immunotherapy. The results of this comparison will enhance our understanding of the immunological landscape within PDAC and its relevance to therapeutic outcomes.

### 2.11 Statistical analysis

Categorical variables were compared using either the chi-square test or Fisher’s exact test. Continuous variables were assessed using the t-test, Mann–Whitney U test, or Kruskal–Wallis rank test. Survival curves were constructed with the Kaplan–Meier method and compared using the log-rank test. Univariate Cox proportional hazards regression analysis was performed to identify prognostic risk factors, and those factors with a p-value < 0.05 were included in the multivariate Cox proportional hazards regression analysis. A significance level of p < 0.05 was established. All statistical analyses were conducted using SPSS software (version 26.0, SPSS Inc., Chicago, IL, USA). Survival curves were generated, and bioinformatic analyses were performed using R software (version 4.4.0).

## 3 Results

### 3.1 Clinicopathological characteristics of patients

**Figure 1:**
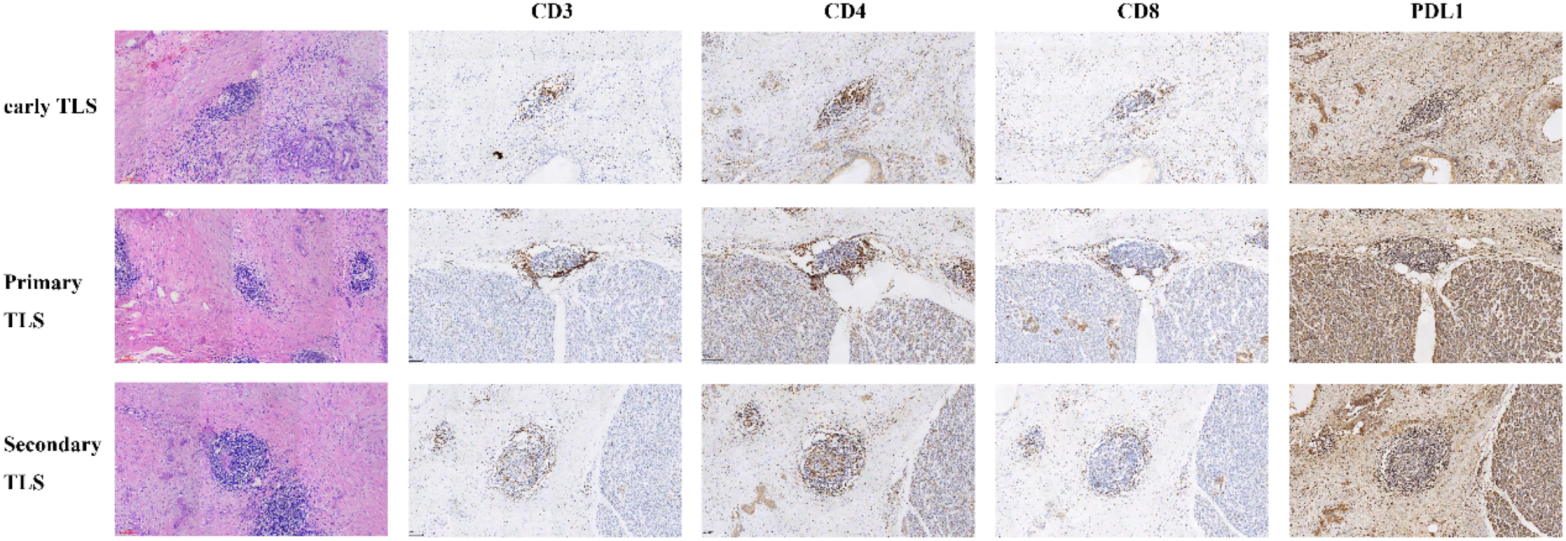
Different maturity of tertiary lymphoid structures (TLSs) identified through hematoxylin and eosin (H&E) staining and the immunohistochemistry of immune cell in corresponding maturity tertiary lymphoid structures.

In the clinical cohort, we analyzed 65 patients diagnosed with PDAC who underwent surgical treatment at our institution. The demographic and clinicopathological characteristics of these patients are detailed in **Table 1**. Among them, 23 patients (35.4%) were identified as positive for iTLS, while the remaining 42 were classified as negative. Notably, the iTLS+ group had a significantly higher proportion of patients at earlier TNM stages (**Table 1**). Additionally, representative images of H&E staining and fluorescent immunohistochemistry (IHC) illustrating iTLS in PDAC patients are displayed in **Figure 1**. In the TCGA cohort, a total of 143 patients diagnosed with surgically treated PDAC from TCGA were included in the study. The demographic and clinicopathological characteristics are outlined in **Table 1**. In this cohort, 72 patients (50.3%) tested positive for iTLS+, while 71 were negative (iTLS-) (**Table 2**).

**Table 1:** Characteristic of patients in CQMU cohort.

Variable	Total	iTLS + (n = 23)	iTLS - (n = 42)	p value
Age	59.8 ± 10.2	60.5 ± 8.6	59.4 ± 11.0	0.227
Gender				0.916
Female	26	9	17	
Male	39	14	25	
Surgical type				0.516
PD	46	15	31	
DP	19	8	11	
Tumor size	3.0 ± 1.1	2.6 ± 0.8	3.3 ± 1.1	0.083
CA199	142.5 (69.1-485.4)	142.5 (33.7-272.2)	155.4 (77.8-677.9)	0.859
CEA	2.65 (1.64-4.58)	2.2 (1.5-3.6)	3.1 (1.8-5.4)	0.18
T				0.019
T1	6	2	4	
T2	49	21	28	
T3	8	0	8	
TX	2	0	2	
N				0.158
N0	56	22	34	
N1	7	1	6	
NX	2	0	2	
Tumor differentiation				0.719
Well	3	2	1	
Moderate	34	12	22	
Poor	25	8	17	
Unkown	3	1	2	
AJCC stage				0.033
IA	6	2	4	
IB	44	20	24	
IIA	6	0	6	
IIB	7	1	6	
Unkown	2	0	2	

PD, pancreaticoduodenectomy; DP, distal pancreatectomy.

**Table 2:** Characteristic of patients in TCGA cohort.

Variable	Total	iTLS + (n = 72)	iTLS - (n = 71)	p value
Age	64.4 ± 10.9	65.0 ± 10.9	63.8 ± 10.8	0.522
Gender				0.211
Female	64	30	34	
Male	79	42	37	
T				0.769
T1	4	2	2	
T2	15	7	8	
T3	120	61	59	
T4	3	1	2	
TX	1	1	0	
N				0.394
N0	36	20	16	
N1	106	52	54	
NX	1	0	1	
Tumor residual				0.863
R0	81	39	42	
R1	46	25	21	
R2	5	3	2	
Unkown	11	5	6	
Tumor differentiation				0.566
Well	20	11	9	
Moderate	82	42	40	
Poor	41	19	22	
AJCC stage				0.48
I	11	8	3	
IIA	23	11	12	
IIB	104	50	52	
III	3	1	2	
IV	3	1	2	
Unkown	1	1	0	

### 3.2 Association between TLS and survival

**Figure 2:**
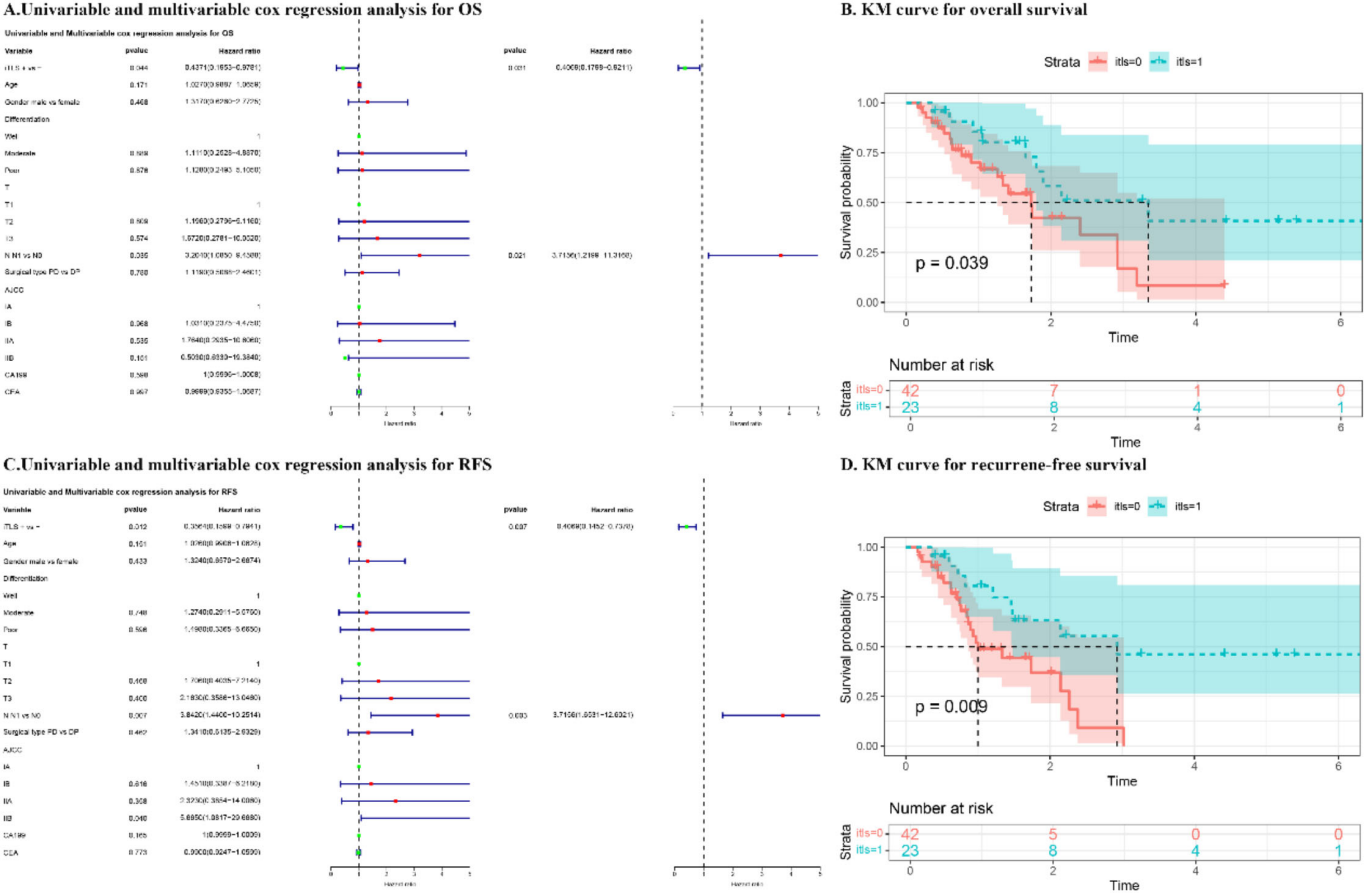
The results of univariate and multivariate cox regression analysis and Kaplan-Meier (KM) curve for overall survival (OS) and recurrence-free survival (RFS) of patients in CQMU. **(A)** Univariable and multivariable cox regression analysis for OS. **(B)** KM curve for OS. **(C)** Univariable and multivariable cox regression analysis for RFS. **(D)** KM curve for RFS.

The findings from both univariate and multivariate analyses, along with the survival curves for patients in the clinical cohort, are presented in **Figure 2**. The univariate analysis demonstrated that the presence of iTLS was associated with improved overall survival (OS) (p = 0.044), while N1 classification was linked to worse OS (p = 0.035). This positive relationship between iTLS and OS remained significant in the multivariate analysis, adjusting for other covariates (HR: 0.407, 95% CI: 0.180-0.921, p = 0.031). Additionally, iTLS was correlated with better recurrence-free survival (RFS) in the univariate analysis (p = 0.012). Conversely, both N1 classification and AJCC IIB stage were associated with reduced OS (p = 0.0007 and p = 0.040, respectively). The prognostic significance of iTLS persisted in the multivariate analysis (HR: 0.407, 95% CI: 0.145-0.738, p = 0.007).

**Figure 3:**
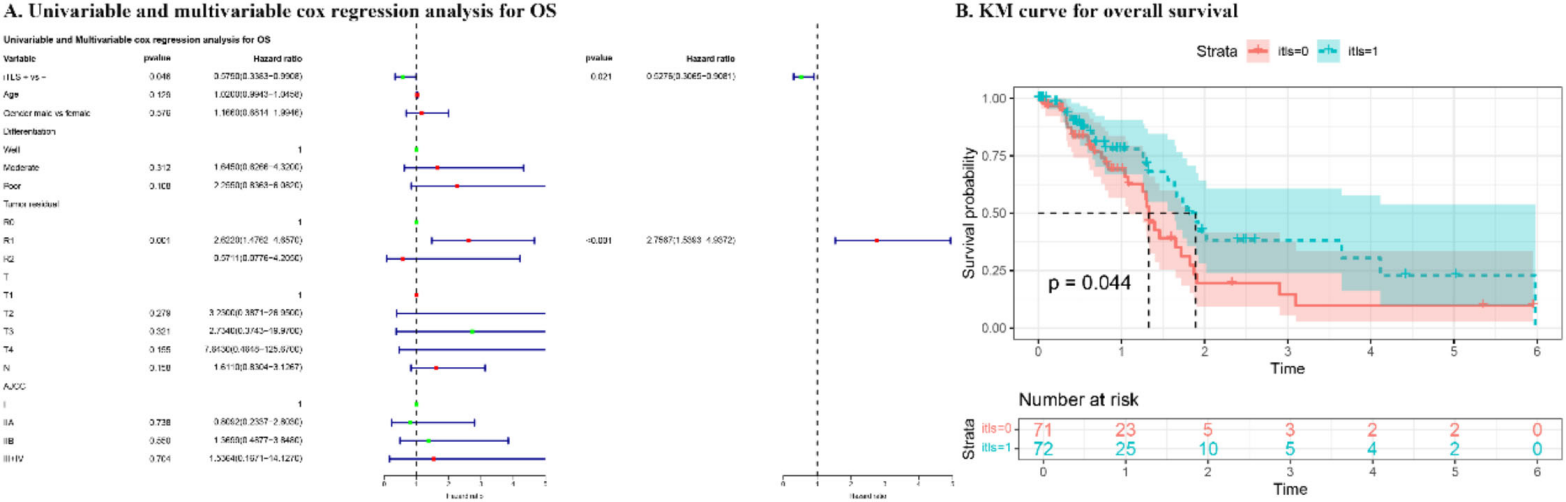
The results of univariate and multivariate cox regression analysis and KM curve for overall survival of patients in TCGA cohort.

The outcomes of both univariate and multivariate analyses and survival curves for patients in the TCGA cohort are presented in **Figure 3**. In the univariate analysis, iTLS+ was associated with improved OS (p = 0.046), while R1 resection was associated with worse OS (p = 0.001). This prognostic significance of iTLS+ remained significant even after adjusting for other factors in the multivariate analysis (HR: 0.528, 95% CI: 0.307-0.908, p = 0.021).

### 3.3 Differentially expressed genes are associated with multiple biological functions

**Figure 4:**
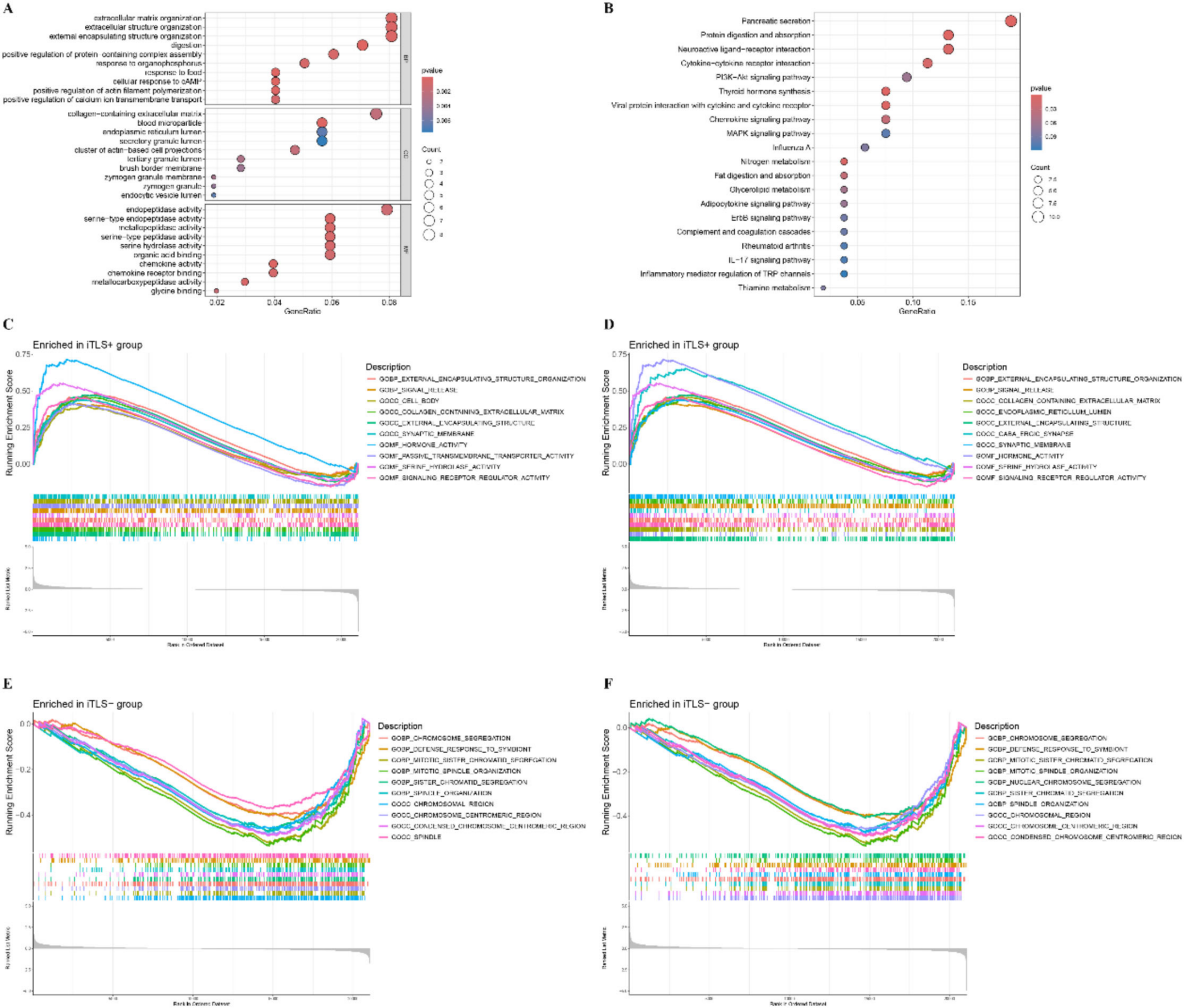
Function annotation for differentially expressed genes (DEGs) related to risk score. **(A)** Functional annotation for DEGs using GO enrichment analysis. The size of the plots represented the number of genes enriched. The pathways were grouped by cellular component (CC), molecular function (MF) and biological process (BP). **(B)** Functional annotation for DEGs using KEGG enrichment analysis. The size of the plots represented the number of genes enriched. **(C, D)** Gene sets enrichment analysis (GSEA) in TLS+ group by GO and KEGG. **(E, F)** Gene sets enrichment analysis (GSEA) in TLS- group by GO and KEGG.

The differential expression genes (DEGs) between the TLS+ and TLS- groups are illustrated in the Volcano and heat maps shown in **Supplementary Figures S1** and **S2**. We observed enrichment in biological processes such as extracellular matrix organization, extracellular structure organization, and external encapsulating structure organization. In terms of cellular components (CC), enrichment was noted in collagen-containing extracellular matrix, blood microparticles, and the endoplasmic reticulum lumen. For molecular functions (MF), key activities included endopeptidase activity, metallopeptidase activity, and serine hydrolase activity (**Figure 4A**). KEGG pathway analysis indicated substantial enrichment in pancreatic secretion, protein digestion and absorption, and neuroactive ligand-receptor interactions (**Figure 4B**). To further explore the differentially active pathways between the TLS+ and TLS- groups, we conducted gene set enrichment analysis (GSEA). The results showed that the TLS+ group exhibited enrichment in signal transduction pathways related to the extracellular matrix, synaptic membranes, passive transmembrane transporter activity, and signaling receptor regulation (**Figures 4C, D**). In contrast, the TLS- group demonstrated significant enrichment in mitotic pathways, including chromosome segregation, defense responses to symbionts, mitotic sister chromatid segregation, mitotic spindle organization, and centromeric regions (**Figures 4E, F**).

### 3.4 Analysis of immune components in TLS+ and TLS- groups

**Figure 5:**
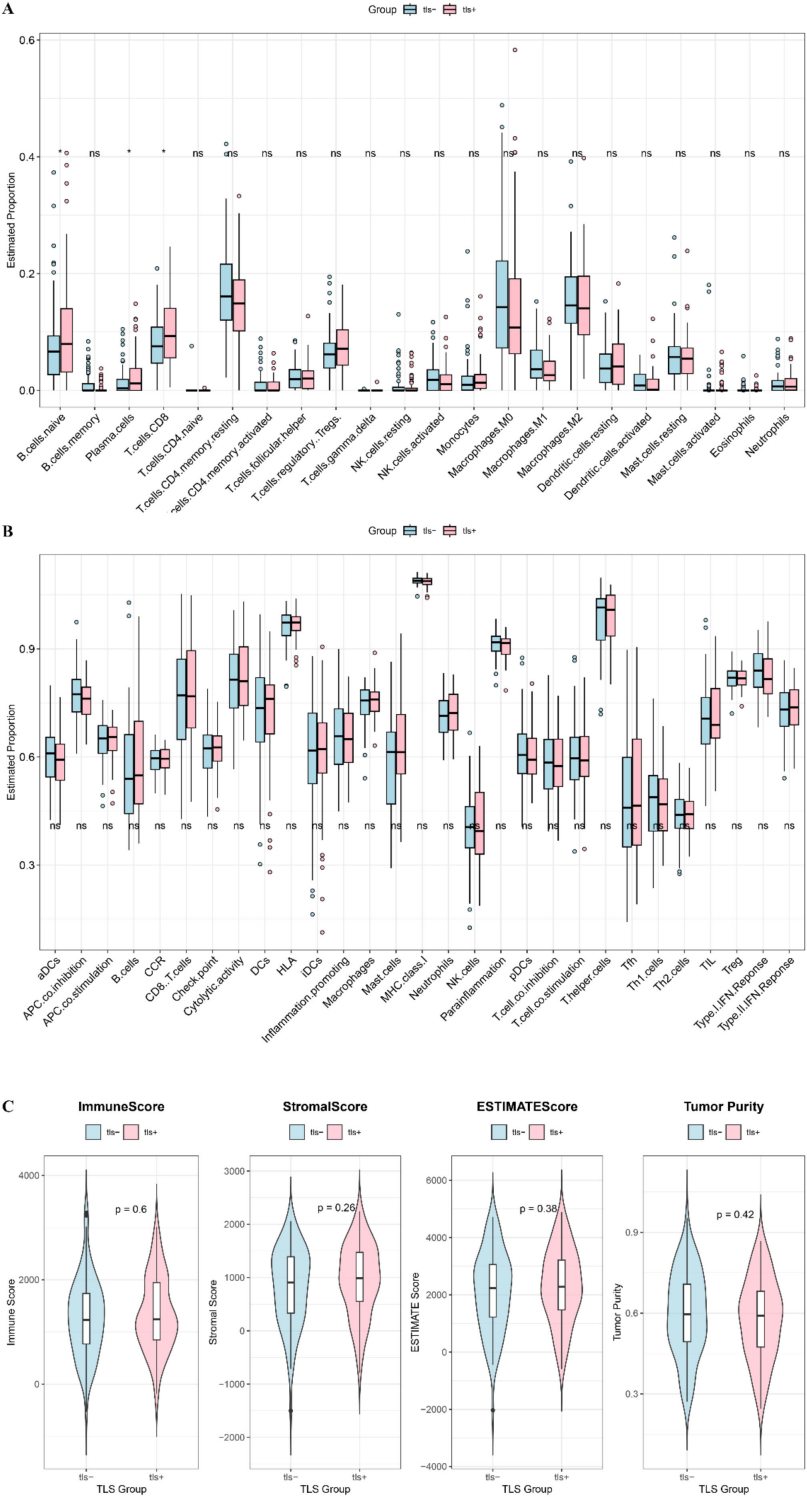
Immune components between high and low risk groups. **(A)** CIBERSORT visualized the infiltration difference of immune cells between TLS+ and TLS- groups. **(B)** Enrichment difference heat map of immune components and immune cells analyzed by ssGSEA method between TLS+ and TLS- groups. **(C)** The immune score, ESTIMATE score, stromal score and tumor purity in the TLS+ and TLS- groups.

To investigate the relationship between the presence of TLS and the regulation of the immune microenvironment, this study assessed the differences in immune cell and component scores between the TLS+ and TLS- groups. CIBERSORT analysis revealed that levels of B cells, T cells, and plasma cells were significantly elevated in the TLS+ group (**Figure 5A**). However, ssGSEA analysis indicated that the immune indicators in the TLS+ group were comparable to those in the TLS- group (**Figure 5B**). Additionally, ESTIMATE analysis showed that the ESTIMATE score, immune score, stromal score, and tumor purity were similar across both groups (**Figure 5C**). These findings highlight the distinct immune characteristics between the TLS+ and TLS- groups, with the higher infiltration of immune components in the TLS+ group suggesting enhanced anti-tumor immune activity.

### 3.5 Differences in mutation characteristics between TLS+ and TLS- groups

**Figure 6:**
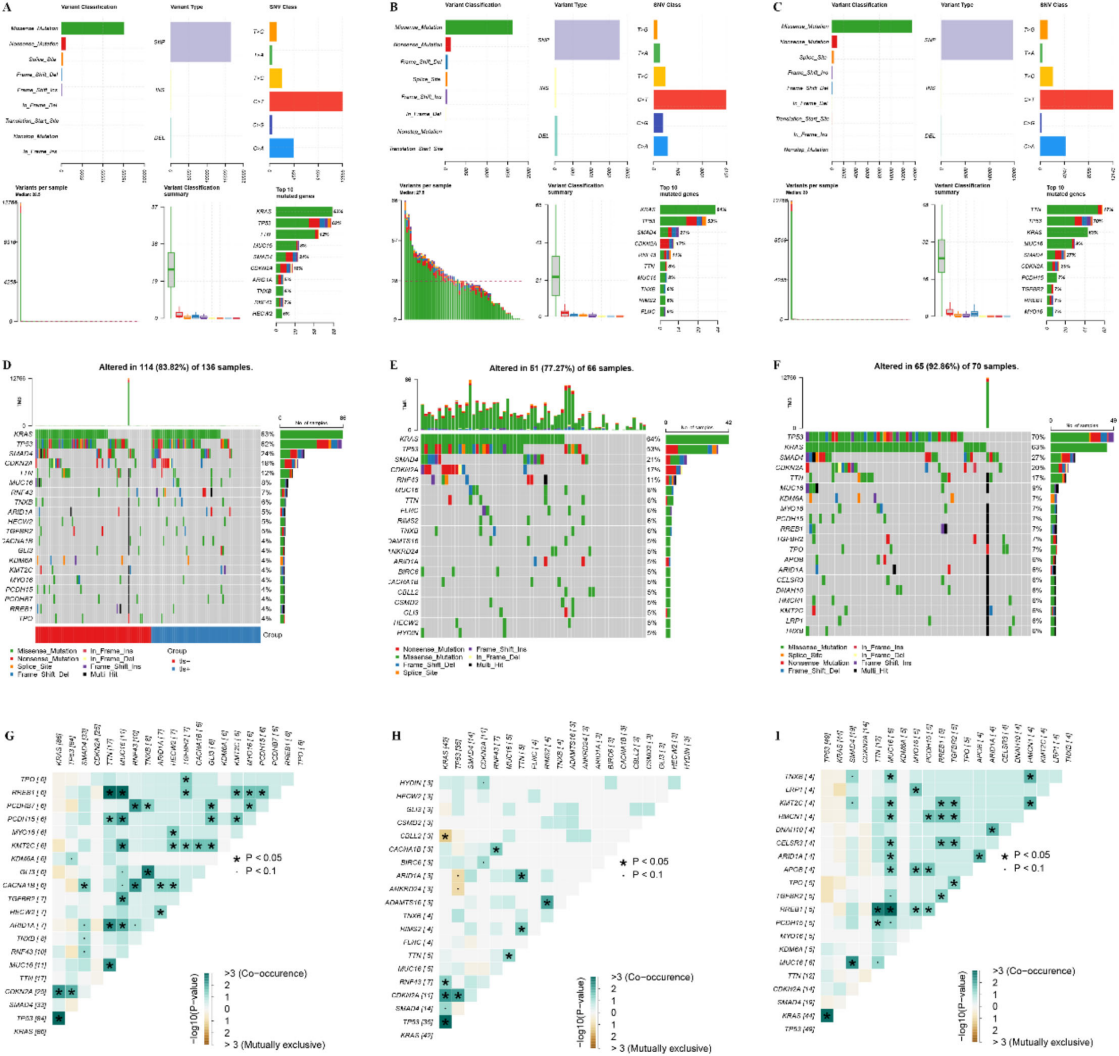
Analysis of genomic mutation frequency in TLS+ and TLS- groups. **(A–C)** Statistics of high-frequency mutation genes, mutation sites, and mutation types in the whole group, TLS+ group, and TLS- group. **(D–F)** The waterfall diagram of the mutation frequency top20 gene in the whole group, TLS+ group, and TLS- group group. **(G–I)** Co-mutation and mutually exclusive mutation map of top20 gene in the whole group, TLS+ group, and TLS- group.

We conducted an analysis of the top 20 genes based on mutation frequency in the TLS+ and TLS- groups. Notable differences were observed in the mutation profiles of these groups, with variations in both the identity of the top mutated genes and their respective mutation frequencies. Significantly, the frequency of TP53 mutations was markedly higher in the TLS- group compared to the TLS+ group (**Figure 6A–F**). Additionally, the co-mutation and mutually exclusive mutation mapping revealed that the TLS- group exhibited a greater proportion of both co-mutations and mutually exclusive mutations relative to the TLS+ group (**Figure 6G–I**). These findings underscore the heterogeneity in gene mutation patterns between the TLS+ and TLS- cohorts, suggesting that these variations may contribute to the differential prognostic outcomes observed in the samples.

### 3.6 Sensitivity analysis of small molecule drugs in the TLS+ and TLS- groups

**Figure 7:**
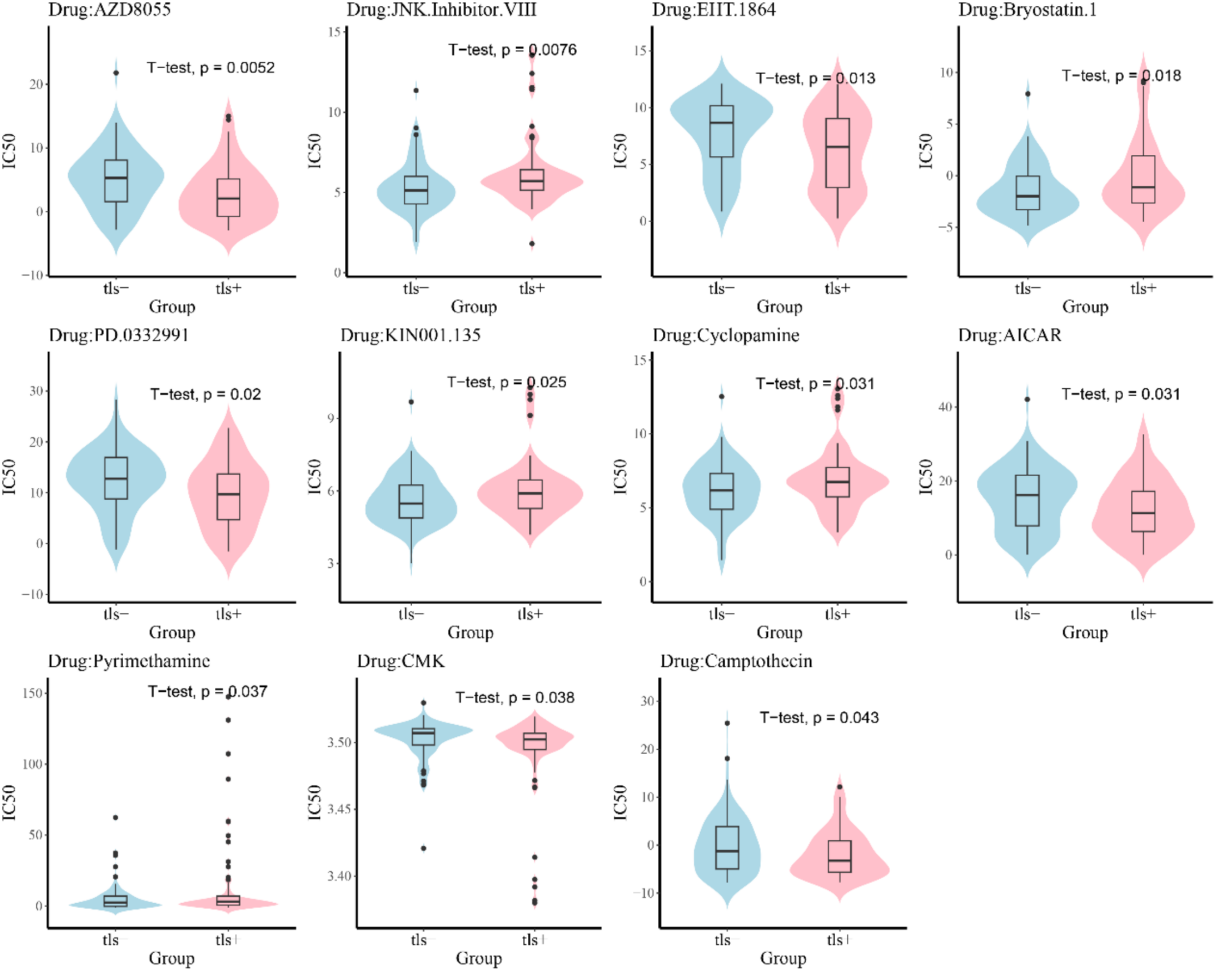
Diference evaluation of small molecule drug sensitivity of samples.

To elucidate the differences in chemotherapy sensitivity between the TLS+ and TLS- groups within the prognostic model, this study analyzed the IC50 values of various chemotherapeutic agents on samples from both groups. The results revealed that six chemotherapy drugs—AZD8055, EHT.1864, PD.0332991, AICAR, CMK, and Camptothecin—exhibited significantly higher IC50 values in the TLS- group, indicating increased resistance to these agents. Conversely, five drugs—JKN.Inhibitor.VIII, Bryostatin.1, KIN001.135, Cyclopamine, and Pyriethamine—showed significantly elevated IC50 values in the TLS+ group (**Figure 7**). These findings suggest a differential resistance profile to specific chemotherapeutic agents between the two groups, highlighting the potential impact of the tumor immune microenvironment on treatment efficacy. Understanding these variations in drug sensitivity could be critical for optimizing individualized treatment strategies in patients with PDAC.

### 3.7 Response to the immunotherapy in the TLS+ and TLS- groups

**Figure 8:**
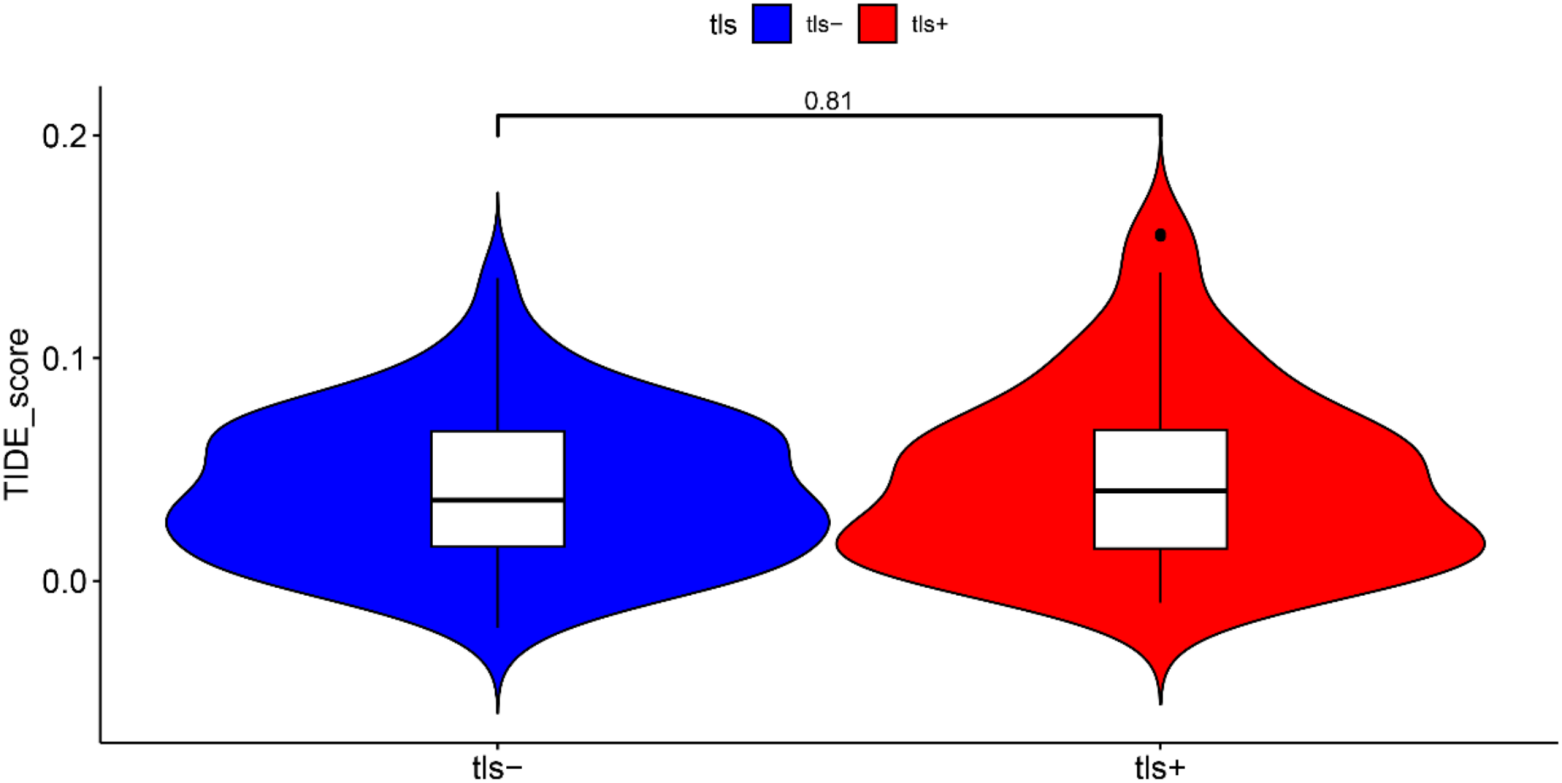
The response to immunotherapy evaluated by TIDE algorithm.

We investigated the differences in immunotherapy response between the TLS+ and TLS- groups within the prognostic model using the TIDE algorithm. Our analysis revealed that the TIDE scores were comparable between the two groups, indicating that the presence of tertiary lymphoid structures did not significantly influence the predicted response to immunotherapy. This finding suggests that, despite the distinct immune microenvironments represented by TLS, the overall effectiveness of immunotherapeutic approaches may not vary substantially based on TLS status (**Figure 8**).

## 4 Discussion

This study employed both a local clinical cohort and data from TCGA to investigate the relationship between the presence of TLS and prognosis in patients with PDAC. Additionally, we performed comprehensive bioinformatics analyses using TCGA data to compare immune cell composition, gene mutations, signaling pathways, and potential drug sensitivities between TLS+ and TLS- PDAC patients. Our results revealed that patients with TLS+ exhibited significantly different prognoses compared to their TLS- counterparts. Furthermore, we observed marked differences between the two groups regarding immune cell composition, gene mutations, signaling pathways, and drug sensitivity profiles.

Our findings indicate that the presence of TLS is associated with improved overall survival (OS) and recurrence-free survival (RFS) in patients with PDAC, aligning with previous research (12–15). This suggests that TLS can serve as a positive prognostic indicator for these patients. Our study provides a more precise classification of TLS in PDAC by directly assessing their presence through histopathological examination, unlike previous approaches that relied solely on TLS-related gene expression profiles in TCGA datasets (17, 18). This methodological advancement minimizes biases from transcriptional heterogeneity and non-TLS-specific gene expression, thereby enhancing the reliability of subsequent analyses. Consistent with prior reports, we confirmed that TLS+ cases exhibited higher immune cell infiltration, likely due to the chemokine-mediated recruitment of lymphocytes (19). Notably, our histology-based approach revealed nuanced spatial distributions of TLS that gene expression alone could not resolve, offering deeper insights into their functional role in tumor-immune interactions.

In terms of biological processes, the differences between the groups primarily focused on tumor proliferation. In the TLS- group, genes with high expression levels were enriched in functions related to tumor proliferation, including spindle organization and chromosome segregation. This suggests that tumor cells in the TLS- group exhibit more active proliferation compared to those in the TLS+ group, which may contribute to the poorer prognosis observed in TLS- patients (20).

In examining immune cell composition, CIBERSORT analysis demonstrated that the TLS+ group exhibited significantly higher levels of immune cell populations, including B cells, T cells, and plasma cells, compared to the TLS- group. This increase in immune cell density within the tumor microenvironment of the TLS+ group suggests a more robust immune response, which is likely to contribute to the improved prognosis observed in these patients (6, 21). The presence of these immune cells may enhance anti-tumor activity, leading to better clinical outcomes. However, despite the differences in immune cell quantities, our analysis revealed no significant variation in TIDE scores between the two groups. This finding implies that the responsiveness to immunotherapy may be similar for both TLS+ and TLS- patients. The lack of difference in TIDE scores could reflect the intrinsic challenges associated with treating PDAC, as this type of cancer is often resistant to immunotherapeutic agents (22). The limited effectiveness of such therapies in PDAC may stem from several factors, including the immunosuppressive tumor microenvironment and the unique biological characteristics of the tumor itself, which often impede the activation and function of immune cells. These insights highlight the complexity of the immune landscape in PDAC and underscore the need for more targeted therapeutic strategies that can effectively engage the immune system, particularly in the context of a tumor microenvironment that poses significant challenges to traditional immunotherapy approaches.

In the context of gene mutations, notable differences were observed in the frequencies of critical genes (23), including TP53, KRAS, SMAD4, and CDKN2A, between the two groups, with TP53 mutations showing a variation of up to 16%. A previous study reported that cases with TP53 alterations had significantly worse immune status (24). In pancreatic cancer, such gene mutations have significant implications for patient prognosis (23), and emerging therapies targeting specific mutations have been shown to improve survival outcomes (25). Additionally, we also found differences in drug resistance between TLS+ and TLS- patients, suggesting that TLS status could guide drug selection to improve outcomes (26).

Several limitations should be considered. First, this study is a single-center retrospective analysis, which is inherently susceptible to biases. Additionally, our findings require validation through larger-scale studies to establish their generalizability. The adequacy of using a single tissue slice to accurately reflect the status of TLS is also questionable, highlighting the need for more precise assessment methods. Furthermore, the pancreatic tumor microenvironment contains various other cell types, including a significant number of fibroblasts and their secreted products, which are linked to both prognosis and TLS formation (27–29). Thus, concentrating solely on immune cell analysis does not provide a comprehensive understanding of the differences between the tumor microenvironments of the TLS+ and TLS- groups. Despite these limitations, our study is the first to illustrate the prognostic significance of iTLS in patients with PDAC.

## 5 Conclusion

This study highlights the significant prognostic implications of iTLS in patients with PDAC. Our results demonstrate that the presence of iTLS is associated with improved OS and RFS, indicating a favorable immune response within the tumor microenvironment. The differential analysis between patients with iTLS+ and iTLS- reveals critical insights into immune cell composition and gene mutation patterns, further supporting the notion that iTLS presence contributes positively to patient outcomes. While this study is limited by its retrospective design and single-center cohort, the findings underscore the potential of iTLS as a biomarker for prognosis and a target for future therapeutic strategies in managing PDAC.

## Data Availability

The datasets presented in this study can be found in online repositories. The names of the repository/repositories and accession number(s) can be found in the article/**Supplementary Material**.
