# Supplementary material for: Investigating the correlation between tertiary lymphoid structures and clinical outcomes in pancreatic ductal adenocarcinoma: insights into tumor immunology
Source: Front Oncol. 2025 Jun 26;15:1569947. doi: 10.3389/fonc.2025.1569947 (PMC12240791; doi:10.3389/fonc.2025.1569947)
Supplement: Supplementary file 1 [file DataSheet1.docx]

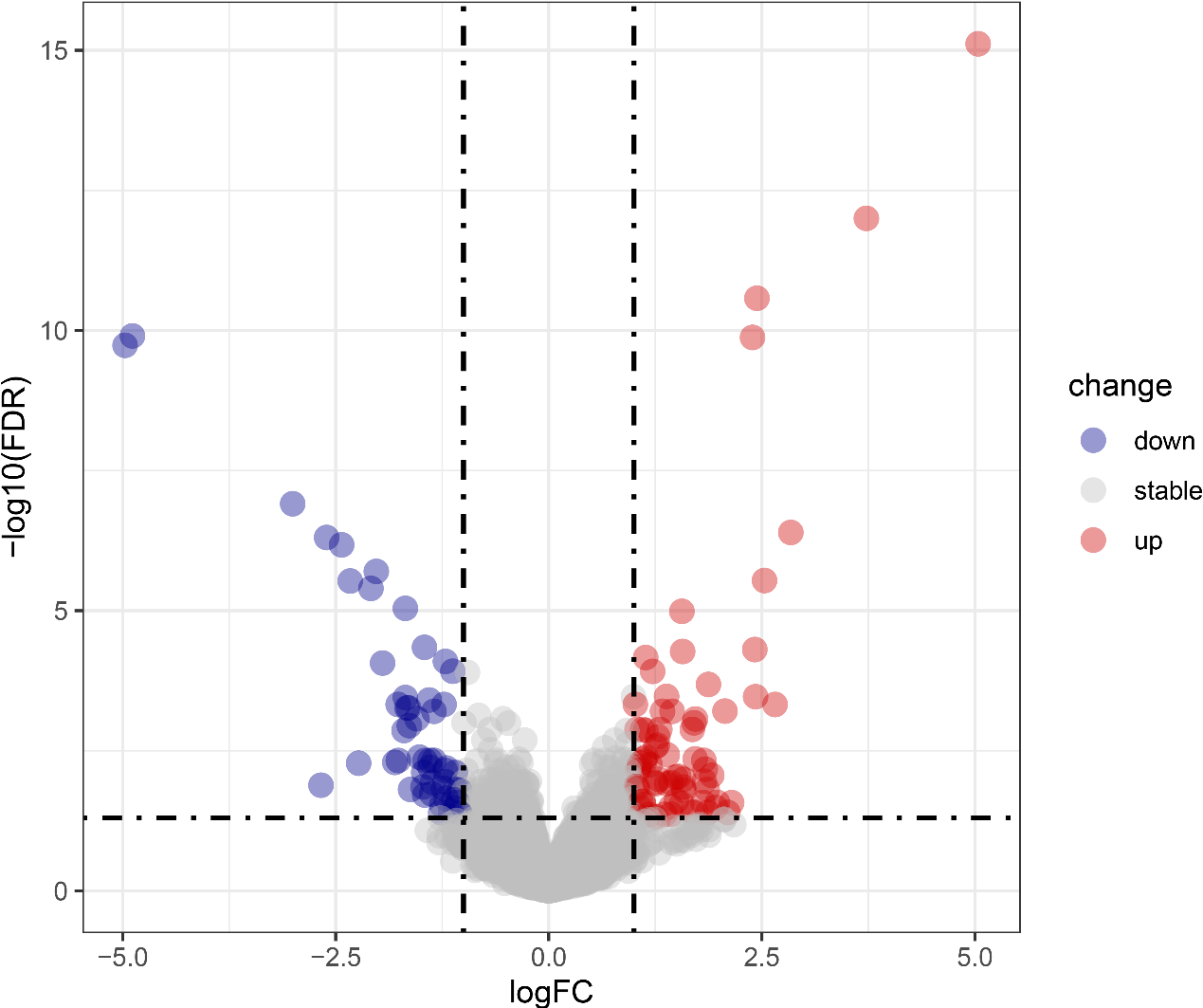
 Figure S1. The volcano maps of differential expressed genes (DEGs).


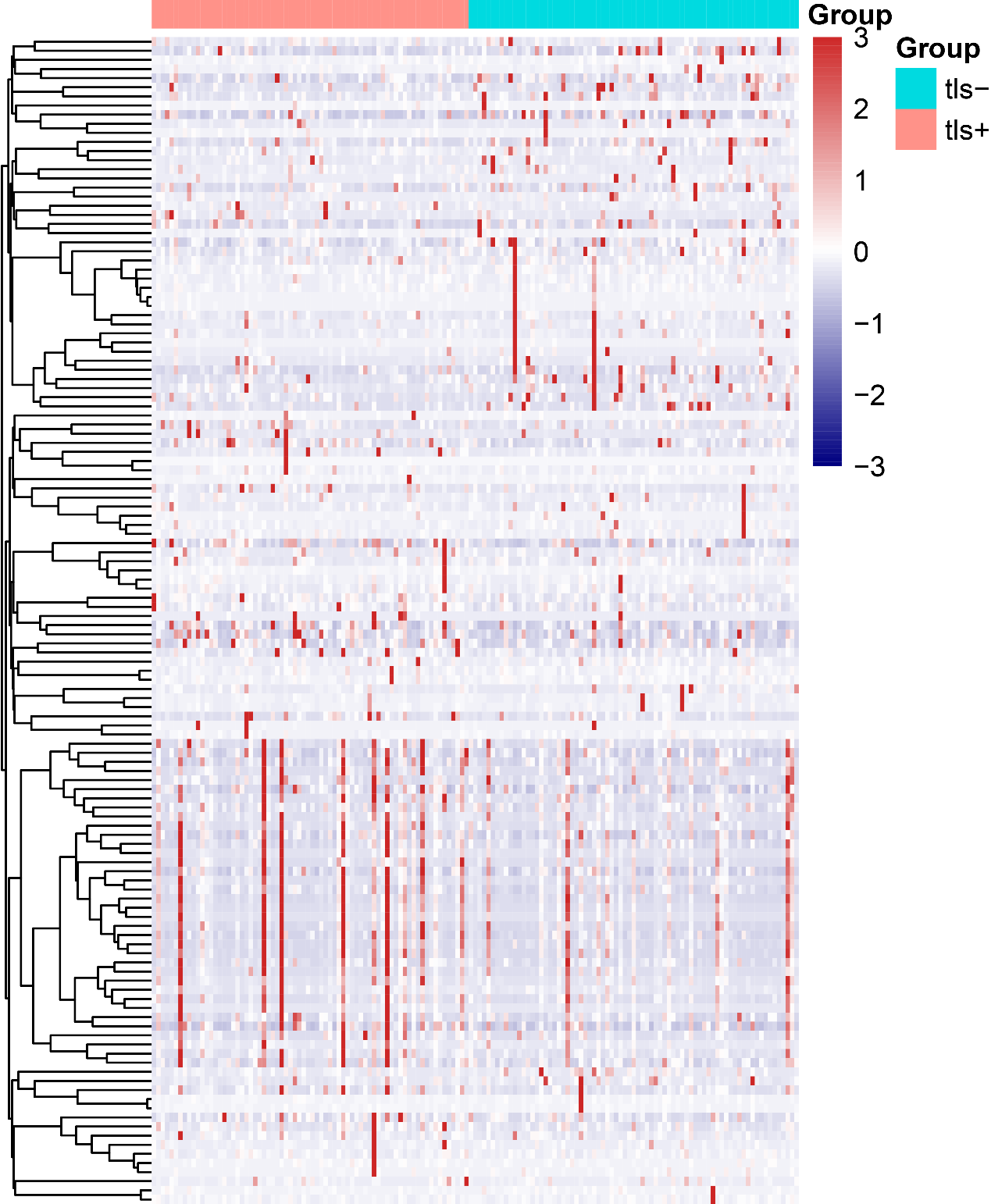


Figure S2. The heatmap of differential expressed genes (DEGs).
